# A Paramagnetic Metal‐Organic Framework Enhances Mild Magnetic Hyperthermia Therapy by Downregulating Heat Shock Proteins and Promoting Ferroptosis via Aggravation of Two‐Way Regulated Redox Dyshomeostasis

**DOI:** 10.1002/advs.202306178

**Published:** 2023-12-31

**Authors:** Yi Wang, Zelong Chen, Jiahui Li, Yafei Wen, Jiaxuan Li, Yinghua Lv, Zhichao Pei, Yuxin Pei

**Affiliations:** ^1^ College of Chemistry and Pharmacy Northwest A&F University Yangling Shaanxi 712100 P. R. China

**Keywords:** ferroptosis, heat shock proteins, mild magnetic hyperthermia therapy, paramagnetic metal‐organic framework, redox dyshomeostasis

## Abstract

Mild magnetic hyperthermia therapy (MMHT) holds great potential in treating deep‐seated tumors, but its efficacy is impaired by the upregulation of heat shock proteins (HSPs) during the treatment process. Herein, Lac‐FcMOF, a lactose derivative (Lac‐NH_2_) modified paramagnetic metal‐organic framework (FcMOF) with magnetic hyperthermia property and thermal stability, has been developed to enhance MMHT therapeutic efficacy. In vitro studies showed that Lac‐FcMOF aggravates two‐way regulated redox dyshomeostasis (RDH) via magnetothermal‐accelerated ferricenium ions‐mediated consumption of glutathione and ferrocene‐catalyzed generation of ∙OH to induce oxidative damage and inhibit heat shock protein 70 (HSP70) synthesis, thus significantly enhancing the anti‐cancer efficacy of MMHT. Aggravated RDH promotes glutathione peroxidase 4 inactivation and lipid peroxidation to promote ferroptosis, which further synergizes with MMHT. H22‐tumor‐bearing mice treated with Lac‐FcMOF under alternating magnetic field (AMF) demonstrated a 90.4% inhibition of tumor growth. This work therefore provides a new strategy for the simple construction of a magnetic hyperthermia agent that enables efficient MMHT by downregulating HSPs and promoting ferroptosis through the aggravation of two‐way regulated RDH.

## Graphical abstract

A paramagnetic metal‐organic framework (Lac‐FcMOF) enhances mild magnetic hyperthermia therapy by downregulating heat shock proteins (HSPs) and promoting ferroptosis through aggravation of two‐way regulated redox dyshomeostasis.

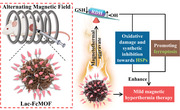

## 1 Introduction

Mild magnetic hyperthermia therapy (MMHT) offers distinct advantages in tissue penetration depth over photothermal therapy (PTT).^[^ ^1^, ^2^, ^3^ ^]^ However, the therapeutic efficacy of MMHT is severely limited due to thermo‐resistance caused by the increase in heat shock proteins (HSPs) as a result of the treatment.^[^ ^4^ ^]^ To date, two methods have been reported to downregulate HSPs to enhance MMHT. The first method is to inhibit the biosynthesis of HSPs using inhibitors, siRNA, or reducing the energy supply (e.g., adenosine triphosphate (ATP)). The second method involves inducing oxidative damage toward HSPs by increasing ∙OH via the peroxidase‐like property of ferrites.^[^ ^5^, ^6^, ^7^, ^8^, ^9^ ^]^ However, these approaches lead to complex construction processes and compositions of therapeutic systems. Furthermore, cellular redox homeostasis restricts the oxidative damage to HSPs.^[^ ^10^ ^]^

Cellular redox homeostasis is a dynamic process that ensures the intracellular balance between reducing and oxidizing reactions, and plays a key role in cancer cell survival and rapid growth.^[^ ^11^ ^]^ Disrupting the redox homeostasis, also known as redox dyshomeostasis (RDH), is a potential anti‐tumor strategy that could inhibit the proliferation of tumor cells.^[^ ^12^, ^13^ ^]^ Cancer specific cellular redox homeostasis contains a high concentration of glutathione (GSH), which serves as the primary reducing agent for scavenging intracellular reactive oxygen species (ROS) and restricts ROS‐mediated downregulation of HSPs.^[^ ^14^, ^15^, ^16^ ^]^ It has been pointed out that compared with only increasing ROS, a two‐way regulation strategy that simultaneously decreases intracellular GSH and elevates ROS can induce a faster and higher degree of RDH, resulting in more severe oxidative damage toward biomacromolecules, including HSPs.^[^ ^13^, ^17^, ^18^ ^]^ The increase in temperature induced by MMHT has the potential to accelerate redox reaction‐mediated GSH consumption as well as Fenton reaction rate to further aggravate two‐way regulated RDH and achieve enhanced HSP oxidative damage. Furthermore, RDH also causes oxidative damage to the mitochondria, which subsequently reduces ATP and thus inhibits HSP synthesis.^[^ ^19^, ^20^ ^]^ Therefore, developing a structurally simple and easily constructed magnetic hyperthermia agent capable of two‐way regulated RDH holds significant potential in achieving efficient MMHT.

At present, therapeutic systems capable of downregulating HSPs for MMHT are based on ferrites that require complex construction processes and multiple components.^[^ ^5^, ^7^, ^8^, ^19^, ^21^, ^22^, ^23^, ^24^, ^25^ ^]^ In search for a more suitable ferrite candidate, metal‐organic frameworks (MOFs) stand out due to their intrinsic superiorities such as multifunctionality, porosity, customizable size, ease of synthesis and modifiability,^[^ ^26^, ^27^, ^28^ ^]^ and the possibility of achieving two‐way regulated RDH by selecting suitable metal ions and organic ligands.^[^ ^29^, ^30^ ^]^ To the best of our knowledge, no MOFs capable of two‐way regulated RDH have been reported as magnetic hyperthermia agents.

**Scheme 1:**
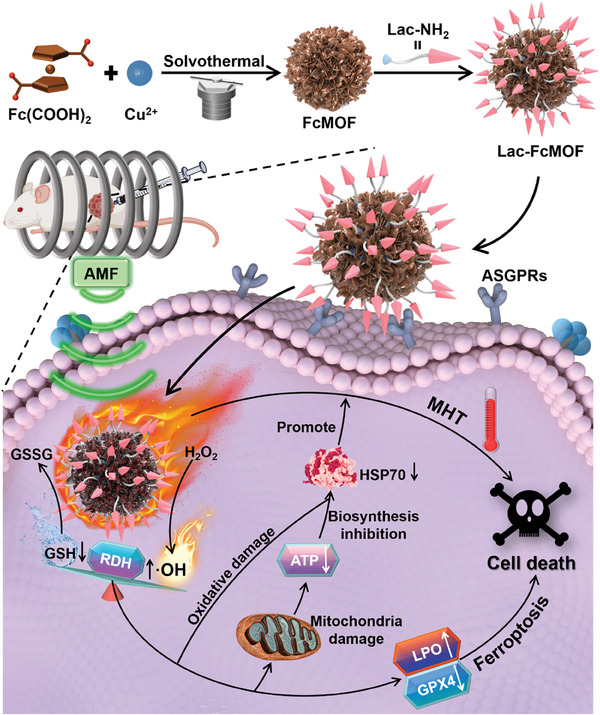
Schematic illustration of the fabrication and the anti‐cancer mechanism of Lac‐FcMOF.

Considering the reductive nature of ferrocene,^[^ ^31^, ^32^, ^33^ ^]^ herein, a paramagnetic FcMOF containing both Fc(COOH)_2_ and [Fc(COOH)_2_]^+^ was synthesized via a solvothermal method using a reducible organic ligand Fc(COOH)_2_ and CuCl_2_. A lactose derivative (Lac‐NH_2_) was then modified onto the surface of FcMOF (denoted as Lac‐FcMOF) to improve its dispersibility in water and endow it with targetability toward HepG2 cells.^[^ ^31^, ^34^, ^35^, ^36^ ^]^ After internalization into cells, [Fc(COOH)_2_]^+^ in Lac‐FcMOF consumed GSH through a redox reaction. Simultaneously, Fc(COOH)_2_ catalyzed the generation of highly oxidative ∙OH via a Fenton‐like reaction to achieve two‐way regulated RDH, which was further aggravated by the magnetothermal effect of Lac‐FcMOF under an alternating magnetic field (AMF). The aggravated RDH caused oxidative damage to heat shock protein 70 (HSP70) as well as the mitochondria, leading to the inhibition of HSP70 synthesis due to reduction in ATP, which together resulted in significant downregulation of intracellular HSP70 and promotion of MMHT. Additionally, the aggravated RDH promoted ferroptosis, a recently‐discovered type of cell death that is inherently potent in eliminating resistant tumor cells, through increased lipid peroxidation and glutathione peroxidase 4 (GPX4) inactivation, which synergizes with MMHT to induce more efficient tumor cell death (**Scheme** **1**).^[^ ^37^, ^38^, ^39^, ^40^, ^41^, ^42^ ^]^ We believe that the paramagnetic FcMOF provided in this work represents an excellent example of a simple yet efficient magnetic hyperthermia agent with significant potential to improve the therapeutic efficacy of MMHT by aggravating two‐way regulated RDH.

## 2 Results and Discussion

### 2.1 Synthesis and Characterization of Lac‐FcMOF

**Figure 1:**
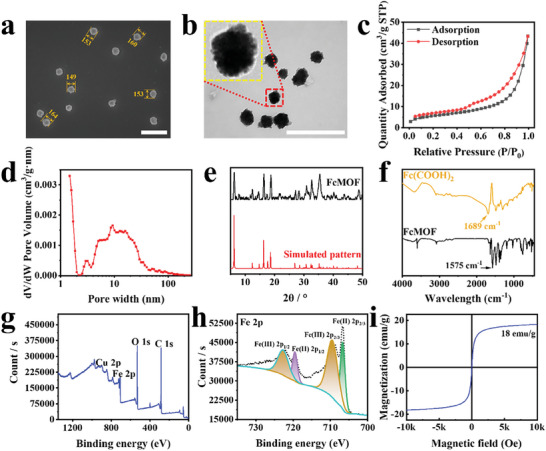
Characterization of FcMOF. a) SEM image of FcMOF. Scale bar: 500 nm. b) TEM image of FcMOF, the nanoparticle in the yellow box is a magnified image of the one in the red box. Scale bar: 500 nm. c) N_2_ adsorption/desorption isotherms of FcMOF. d) Pore width distribution of FcMOF. e) Experimental and simulated XRD patterns of FcMOF. f) FTIR spectra of Fc(COOH)_2_ and FcMOF. g) XPS pattern of FcMOF. h) XPS spectra of Fe 2p orbit for FcMOF. i) Field‐dependent magnetization hysteresis loop of FcMOF at 300 K.

Nanoscale FcMOF was prepared according to previously reported methods with some modifications.^[^ ^43^, ^44^ ^]^ CuCl_2_·2H_2_O, Fc(COOH)_2_ and polyvinylpyrrolidone (PVP) were completely dissolved in N, *N*‐dimethylformamide (DMF), then transferred to a Teflon‐lined autoclave, and heated to 150 °C for 24 h. During this process, FcMOF formed through the coordination between Cu and Fc(COOH)_2_, while PVP regulated the morphology and improved the thermostability and reproducibility.^[^ ^43^, ^45^ ^]^ The morphology and scale of FcMOF were characterized by scanning electron microscopy (SEM) and transmission electron microscopy (TEM), respectively. As shown in **Figure** **1**a,b and Figure S1 (Supporting Information), FcMOF is largely spherical, uniform, with an average size of 156 nm, and has a porous structure. The micro‐mesoporosity of FcMOF was verified using N_2_ adsorption/desorption isotherm (Figure 1c). The N_2_ absorption/desorption curve displayed a type H3 hysteresis loop, indicative of the characteristic mesoporous structure created by plate‐like particles or layers, which is consistent with the TEM images. The BET surface area and pore volume of FcMOF are 19.67 m^2^ g^−1^ and 0.04 cm^3^ g^−1^, respectively. The pore size distribution curve showed that the average pore size of FcMOF is 10.44 nm (Figure 1d), which suggests drug loading potential. Furthermore, the powder X‐ray diffraction (PXRD) pattern of FcMOF showed characteristic peaks at 6.2^◦^, 12.5^◦^, 14.7^◦^, 16.3^◦^, 18.7^◦^, 27.5^◦^, 31.3^◦^, 33.0^◦^, and 35.8^◦^, which align well with the simulated pattern and the patterns of previously reported FMOF‐Co and Zn‐Fc MOF (microscale MOF constructed by the coordination of Fc(COOH)_2_ with Co^2+^ or Zn^2+^) (Figure 1e),^[^ ^43^, ^44^ ^]^ indicating that FcMOF is isostructural to the reported FMOF‐Co and Zn‐Fc MOF and has a highly crystalline structure.

Fourier transform infrared spectroscopy (FTIR) spectra of Fc(COOH)_2_ and FcMOF showed that the C = O stretching frequency of Fc(COOH)_2_ shifted from 1689 to 1575 cm^−1^ after the formation of FcMOF (Figure 1f), which indicates the coordination between the carboxyl of Fc(COOH)_2_ and Cu^2+^.^[^ ^43^ ^]^ The signals of Cu 2p, Fe 2p, O 1s, and C 1s could be observed in the X‐ray photoelectron spectroscopy (XPS) spectrum (Figure 1g), indicating the presence of Cu, Fe, O, and C elements in FcMOF. The two characteristic peaks at 706.8 and 719.5 eV could be ascribed to Fe^2+^, while 709.6 and 722.9 eV peaks could be ascribed to Fe^3+^ (Figure 1h), suggesting that FcMOF possesses the potential of achieving two‐way regulated RDH. Since ferrocenium ion is paramagnetic and used to synthesize magnetic materials, the magnetic property of FcMOF was further investigated.^[^ ^46^, ^47^ ^]^ FcMOF dispersed in water can be attracted very quickly by a magnet (Figure S2, Supporting Information), with the saturation magnetization of FcMOF measured to be 18 emu g^−1^ at 300 K (Figure 1i). Crucially, the coercive force in FcMOF is less than 7 Oe (Figure S3, Supporting Information), suggesting its paramagnetic property, which is beneficial for biomedical uses.^[^ ^48^ ^]^

**Figure 2:**
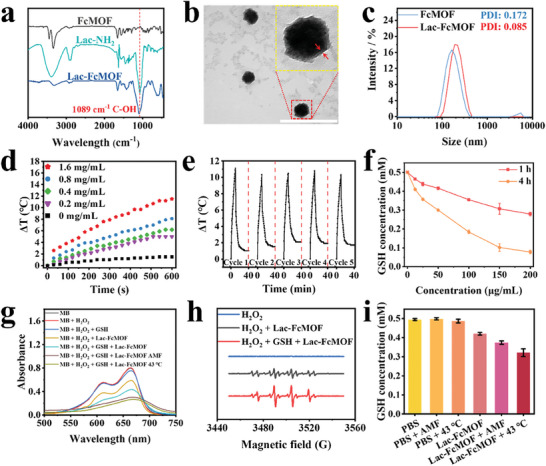
Characterization of Lac‐FcMOF. a) FTIR spectra of FcMOF, Lac‐NH_2_ and Lac‐FcMOF. b) TEM image of Lac‐FcMOF, the nanoparticle in the yellow box is a magnified image of the one in the red box. Scale bar: 500 nm. c) Hydrodynamic diameter distribution and PDI of FcMOF and Lac‐FcMOF. d) Temperature‐time plots for different Lac‐FcMOF concentrations in water under AMF for 10 min. e) Cyclic magnetic heating curve of Lac‐FcMOF (1.6 mg mL^−1^) in water for five cycles under AMF. f) The consumption of GSH by various concentrations of Lac‐FcMOF for different timeframes. Data are shown as the mean ± SD (n = 3). g) UV–vis spectra of MB at pH 5.0 after different treatments for 10 min. The concentration of Lac‐FcMOF was 100 µg mL^−1^, MB was 10 µg mL^−1^, H_2_O_2_ was 10 mM, and GSH was 10 mM. h) ESR spectra of H_2_O_2_ (10 mM), H_2_O_2_ (10 mM) + Lac‐FcMOF (100 µg mL^−1^), and H_2_O_2_ (10 mM) + GSH (10 mM) + Lac‐FcMOF (100 µg mL^−1^) at pH 5.0. i) The consumption of GSH within 10 min by different treatments, the concentration of Lac‐FcMOF was 200 µg mL^−1^. Data are shown as the mean ± SD (n = 3).

Lactose is abundant and easily obtained in nature. Its rich hydroxyl group can improve the hydrophilicity of molecules or materials, and it can target asialoglycoprotein receptors (ASGPRs) overexpressed on the surface of liver tumor cells. To improve the dispersion of FcMOF in water and endow it with liver tumor targetability to reduce the side effects toward normal cells, Lac‐NH_2_, a lactose derivative with an amino terminal, was synthesized. Lac‐NH_2_ was then modified to the surface of FcMOF through non‐covalent bonds, including hydrogen bonds and coordination bonds formed by the interaction between the amino groups at the end of Lac‐NH_2_ with the abundant carboxyl groups and copper ions on FcMOF, resulting in the formation of Lac‐FcMOF. The detailed synthetic route and characterization of Lac‐NH_2_ are shown in Figures S4–S8 (Supporting Information). The FTIR spectra in **Figure** **2**a showed that the C‐OH stretching frequency of Lac‐NH_2_ at 1089 cm^−1^ appeared in Lac‐FcMOF, indicating successful modification. Furthermore, a clear cladding on the surface of FcMOF after the modification with Lac‐NH_2_ is visible in the TEM image in Figure 2b. After the modification with Lac‐NH_2_, the hydrodynamic diameter of Lac‐FcMOF increased to ≈197 nm, which is larger than FcMOF (≈169 nm). Additionally, the Zeta potential changed from −13.7 to −23.1 mV, and the polydispersity index (PDI) changed from 0.172 to 0.085 (Figure S9, Supporting Information; Figure 2c), which is due to the improved hydrophilicity endowed by Lac‐NH_2_ to Lac‐FcMOF resulting in better dispersity in water. Together, these results demonstrated the successful construction of Lac‐FcMOF. The stability of Lac‐FcMOF was tested by dynamic light scattering (DLS) (Figure S10, Supporting Information), where the hydrodynamic diameters of Lac‐FcMOF were shown to be stable in different media within 7 days, indicating good disperse stability. The larger hydrodynamic diameter of Lac‐FcMOF in PBS and complete culture medium compared to that in ultrapure water may be attributed to the high concentrations of salt ions in the media. The increased ionic strength might lead to the shielding of charges on the Lac‐FcMOF surface, thereby reducing the electrostatic repulsion between nanoparticles, which could promote aggregation and consequently increase its hydrodynamic diameter. In addition, complete culture medium contains proteins that can adsorb onto the surface of Lac‐FcMOF to form a protein corona, thereby further increasing its hydrodynamic diameter.^[^ ^49^, ^50^, ^51^ ^]^

Lac‐FcMOF still maintains its paramagnetic property (Figure S11, Supporting Information), although the saturation magnetization is slightly decreased due to the lower FcMOF content in each gram of test sample compared to FcMOF alone.^[^ ^52^, ^53^, ^54^ ^]^

### 2.2 Magnetothermal Conversion Efficiency of Lac‐FcMOF

Magnetothermal conversion efficiency of Lac‐FcMOF was studied by exposing microcentrifuge tubes containing different concentrations of Lac‐FcMOF to an AMF for 10 min and measuring the temperature every 30 s. Higher concentrations of Lac‐FcMOF led to faster and higher temperature increases. At a concentration of 1.6 mg mL^−1^, the temperature had increased by 11.5 °C by the end of the exposure (Figure 2d). The specific loss power (SLP) value of Lac‐FcMOF was calculated to be 226.7 W g^−1^, indicating good magnetothermal conversion efficiency. In addition, the magnetic‐thermal stability of Lac‐FcMOF was investigated. As shown in Figure 2e, during five magnetic field on/off cycles, there was no significant temperature deterioration, indicating good magnetic‐thermal stability. The tissue penetrability of Lac‐FcMOF based MMHT was further investigated and compared with PTT by injecting both agents into the center of pork pieces (1×1×1 cm^3^ and 2×2×2 cm^3^). As shown in Figure S12 (Supporting Information), substantial temperature increases on the pork's surface and center were achieved through the magneto‐thermal effect of Lac‐FcMOF. In contrast, photo‐thermal effect induced by BODIPY, an extensively studied photothermal agent, generated far less temperature increase across the pork. This demonstrates that Lac‐FcMOF based MMHT has superior tissue penetration capabilities compared to PTT and has the potential to treat deep‐seated tumors.

### 2.3 GSH Consumption and Fenton Catalytic Activity of Lac‐FcMOF

GSH consumption of Lac‐FcMOF was assessed using a reduced GSH content assay kit while Fenton catalytic activity of Lac‐FcMOF was assessed using methylene blue (MB) to detect ∙OH.^[^ ^55^, ^56^ ^]^ The concentration of GSH decreased with increasing concentration of Lac‐FcMOF and incubation time (Figure 2f), which can be ascribed to ferricenium ions consuming GSH through redox reactions.^[^ ^31^, ^57^ ^]^ MB degradation by Lac‐FcMOF was not observed at pH 7.4 (Figure S13a, Supporting Information), but was observed in the tumor cell microenvironment mimic (10 mM H_2_O_2_, pH 5.0), as the production of ∙OH through Lac‐FcMOF‐catalyzed Fenton reaction requires a lower pH (Figure 2g).^[^ ^57^, ^58^, ^59^, ^60^ ^]^ The presence of high concentrations of H_2_O_2_ and GSH, which mimics the high level of redox homeostasis in cancer cell, further degrades MB. This could be attributed to the reduction of ferricenium ion to ferrocene by GSH, which in turn produced ∙OH through a Fenton reaction to degrade MB. Electron paramagnetic resonance (ESR) spectroscopy results further confirmed ∙OH generation by Lac‐FcMOF‐catalyzed Fenton reaction in the tumor cell microenvironment mimic, where the typical 1:2:2:1 signal of ∙OH was found (Figure 2h). In addition, applying AMF or 43 °C water bath to Lac‐FcMOF accelerated GSH consumption (Figure 2i) and MB degradation (Figure 2g; Figure S13b, Supporting Information), as the increase in temperature sped up the redox and Fenton reactions. Comparatively, GSH consumption and MB degradation were lower in the AMF treated group as the temperature increase was progressive.

Although XPS analysis showed the coexistence of Cu^+^ and Cu^2+^ within FcMOF (Figure S14a, Supporting Information), Cu in FcMOF needs to be released in order to contribute to the regulation of RDH.^[^ ^61^, ^62^, ^63^ ^]^ Stability studies under different conditions indicated that FcMOF has a low degradation rate, inferred from its small changes in morphology and hydrodynamic diameter, and release Cu very slowly (Figure S14b–d, Supporting Information). Thus, RDH is primarily mediated by ferrocene and ferricenium ion in FcMOF, and the contribution of Cu is very limited.

### 2.4 Cellular Uptake, Targeting and In Vitro Cytotoxicity

**Figure 3:**
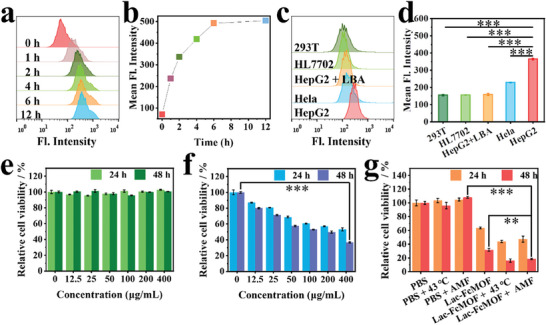
Cellular uptake, targeting, and cytotoxicity. a) Fluorescence intensity of HepG2 cells detected by FCM after incubating with 100 µg mL^−1^ Lac‐FcMOF@FITC at different time points. b) Mean fluorescence intensity of HepG2 cells at various time points in a). c) Fluorescence intensity of different cells detected by FCM after incubation with 100 µg mL^−1^ Lac‐FcMOF@FITC for 4 h. d) Mean fluorescence intensity of different cells in c). Data are shown as the mean ± SD (n = 3) (*** *p* < 0.001). e) Cytotoxicity of Lac‐FcMOF with various concentrations toward HL7702 cells after incubation for 24 and 48 h. f) Cytotoxicity of Lac‐FcMOF at various concentrations toward HepG2 cells after incubation for 24 and 48 h. Data are shown as the mean ± SD (n = 5) (*** *p* < 0.001). g) Relative cell viability of HepG2 cells at 24 and 48 h after different treatments. The concentration of Lac‐FcFMOF was 400 µg mL^−1^, and the processing time of 43 °C water bath and AMF was 10 min. Data are shown as the mean ± SD (n = 5) (** *p* < 0.01, and *** *p* < 0.001).

First, the anti‐tumor effect of FcMOF was investigated. FcMOF showed significant inhibitory efficiency toward HepG2, SKOV3, Hela, and KM‐12 cancer cells (Figure S15, Supporting Information), indicating a broad‐spectrum anti‐tumor effect. Cellular uptake and cancer cell targeting ability of Lac‐FcMOF were then evaluated with flow cytometry (FCM) using Lac‐FcMOF@FITC, which was fabricated through loading FITC (a fluorescence probe) into Lac‐FcMOF by leveraging the porosity of Lac‐FcMOF. With increasing incubation time, the fluorescence intensity of FITC gradually increased and reached saturation at 12 h (**Figure** **3**a and b), demonstrating successful cellular uptake of Lac‐FcMOF with maximum uptake achieved at 12 h. Lactose specifically targets ASGPRs, which are overexpressed on HepG2 cell membranes, based on carbohydrate‐protein interaction. Lactose‐modified nanoparticles can therefore exhibit strong targeting capability for HepG2 cells and reduce side effects. Here, HepG2, Hela (low expression of ASGPRs), lactobionic acid (LBA, an ASGPRs blocker) pre‐treated HepG2, and two normal cell lines with low expression of ASGPRs (293T and HL7702) were incubated with Lac‐FcMOF@FITC to assess its targetability. As shown in Figure 3c and d, compared to 293T, HL7702 and Hela cells, HepG2 cells showed stronger fluorescence intensity, which decreased if they were pre‐treated with LBA. These results indicate that Lac‐FcMOF targets HepG2 cells as expected, due to its Lac‐NH_2_ component displaying specific recognition toward ASGPRs.

To assess the in vitro cytotoxicity of Lac‐FcMOF, MTT assays were conducted using HepG2 and HL7702 cells. The relative cell viability of HL7702 cells was not affected by increasing Lac‐FcMOF concentration (Figure 3e). In contrast, the relative cell viability of HepG2 cells decreased gradually with increasing Lac‐FcMOF concentration (Figure 3f), demonstrating good biocompatibility of Lac‐FcMOF toward normal cells and dose‐dependent cytotoxicity toward cancer cells. Next, the toxicity of Lac‐FcMOF toward HepG2 cells promoted by magnetothermal effect was studied. The viability of HepG2 cells was not affected by either 43 °C water bath or AMF treatment for 10 min on their own. However, viability significantly decreased after incubating HepG2 cells with 400 µg mL^−1^ Lac‐FcMOF before the treatments, with relative cell viability decreasing to ≈19% in both groups (Figure 3g). This result indicates that magnetothermal effect can increase the HepG2 cell killing efficiency of Lac‐FcMOF.

### 2.5 Induction of Intracellular RDH and Downregulation of HSP70

**Figure 4:**
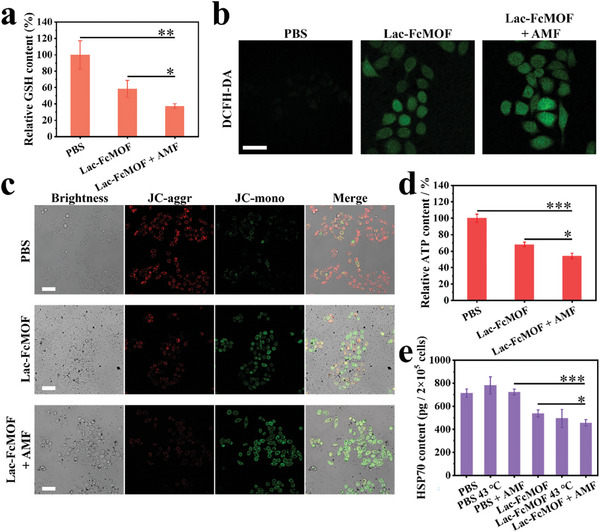
In vitro RDH and HSP70 downregulation. a) Relative GSH content of HepG2 cells after different treatments. b) CLSM images of ROS detected by DCFH‐DA in HepG2 cells after different treatments. The green fluorescence is emitted by DCF (*Ex*: 488 nm, *Em*: 525 nm). Scale bar: 100 µm. c) CLSM images of MMP detected by JC‐10 in HepG2 cells after different treatments. The red fluorescence is emitted by JC‐10 aggregates (*Ex*: 540 nm, *Em*: 590 nm) and the green fluorescence is emitted by JC‐10 monomer (*Ex*: 490 nm, *Em*: 525 nm). Scale bar: 100 µm. d) Relative ATP content of HepG2 cells after different treatments. e) HSP70 content of HepG2 cells after different treatments. The concentration of Lac‐FcFMOF was 400 µg mL^−1^, the incubation time of cells with different treatments was 6 h, and the processing time of AMF and 43 °C water bath was 10 min. Data are shown as the mean ± SD (n = 3) (* *p* < 0.05, ** *p* < 0.01, and *** *p* < 0.001).

Owing to the presence of ferricenium ion and ferrocene, RDH can be induced through the consumption of intracellular GSH and the conversion of endogenous H_2_O_2_ into highly oxidative ∙OH. The relative intracellular GSH content in Lac‐FcMOF treated HepG2 cells was therefore assessed using a reduced GSH content assay kit. A significant decrease in GSH content was observed, which was even further reduced following AMF treatment (**Figure** **4**a). Confocal laser scanning microscopy (CLSM) images revealed increased intracellular ROS, indicated by the green fluorescence, while even stronger fluorescence was observed in cells treated with Lac‐FcMOF + AMF (Figure 4b; Figure S16a, Supporting Information). These results indicate that Lac‐FcMOF effectively consumes GSH and elevates ROS, which can be further enhanced by its magnetothermal effect.

RDH can lead to oxidative damage and dysfunction of the mitochondria.^[^ ^64^ ^]^ Mitochondrial membrane potential (MMP) reversal is a critical marker of mitochondrial damage, which was assessed using the mitochondria‐permeable dye JC‐10. In contrast to the red fluorescence observed in HepG2 cells treated with PBS, green fluorescence was observed in those treated with Lac‐FcMOF, which was further intensified following AMF treatment (Figure 4c). This suggests that Lac‐FcMOF induces depolarization of mitochondrial membrane potential, which is further enhanced by its magnetothermal effect. Additionally, intracellular ATP significantly decreased following Lac‐FcMOF treatment, further confirming mitochondrial dysfunction (Figure 4d). Decreased ATP hinders HSP synthesis, while RDH causes oxidative damage to HSPs, leading to a synergistic HSP downregulation and increased cell sensitivity to heat.^[^ ^6^, ^9^, ^25^ ^]^ As an example, HSP70, a crucial member of HSPs, was analyzed in HepG2 cells after different treatments. AMF treatment alone did not impact HSP70 expression, whereas the PBS group treated with 43 °C water bath saw an upregulation of HSP70 as a heat response. In contrast, all Lac‐FcMOF‐treated groups showed significantly downregulated HSP70, which was further enhanced by AMF treatment (Figure 4e), indicating that Lac‐FcMOF is highly efficient in downregulating HSPs, which is critical in enhancing MMHT efficacy.

### 2.6 Induction of Ferroptosis

**Figure 5:**
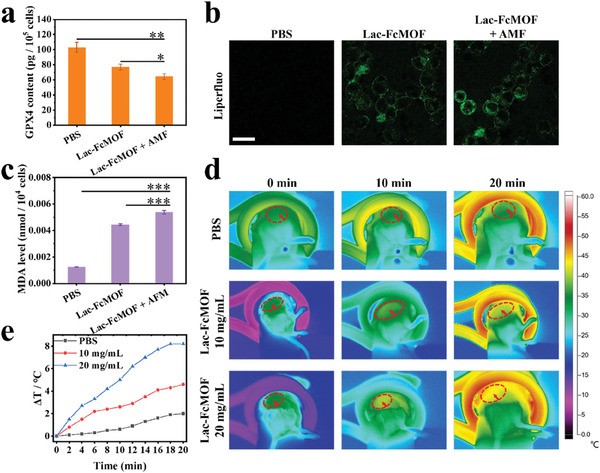
In vitro ferroptosis and in vivo magnetothermal efficiency. a) The GPX4 content of HepG2 cells after different treatments. b) CLSM images of LPO detected by Liperfluo in HepG2 cells after different treatments. The green fluorescence is emitted by Liperfluo (*Ex*: 488 nm, *Em*: 535 nm). Scale bar: 100 µm. c) MDA content of HepG2 cells after different treatments. The concentration of Lac‐FcFMOF was 400 µg mL^−1^, the incubation time of cells with different treatments was 6 h, and the processing time of AMF was 10 min. Data are shown as the mean ± SD (n = 3) (* *p* < 0.05, ** *p* < 0.01, and *** *p* < 0.001). d) Infrared thermal images of mice in AMF after intratumor injection of different concentrations of Lac‐FcMOF. The location of the tumor was marked with red elliptical dashes. e) Time dependent temperature curve of tumor after intratumor injection of different concentrations of Lac‐FcMOF.

Previous work showed that fast GSH consumption leads to ferroptosis through the suppression of GPX4 activity, a critical cofactor in converting toxic lipid peroxides (LPOs) into nontoxic hydroxyl compounds.^[^ ^65^ ^]^ As shown in **Figure** **5**a,b and Figure S16b (Supporting Information), compared to the control, HepG2 cells treated with Lac‐FcMOF displayed significant suppression of GPX4 activity and increase in LPO and malondialdehyde (MDA) (a downstream product of LPO and an important marker of lipid peroxidation). AMF exposure to Lac‐FcMOF treated HepG2 cells further enhanced the above effects.

### 2.7 *T_2_*‐Weighted Magnetic Resonance Imaging of Lac‐FcMOF In Vivo

Due to its magnetic property, Lac‐FcMOF can be used as a potential *T_2_*‐weighted magnetic resonance imaging (MRI) contrast agent. As shown in Figure S17a and b (Supporting Information), the longitudinal relaxivity (r_1_) and transverse relaxivity (r_2_) values were determined to be 1.15 and 24.19 (mM Fe)^−1^ s^−1^. The value of r_2_/r_1_ is 21.03, which is far larger than the required 3 for *T_2_*‐weighted MRI contrast agents.^[^ ^66^ ^]^ When Lac‐FcMOF was used in *T_2_*‐weighted MRI, the negative contrast was observed at an ion concentration range between 0 to 1.6 mM. *T_2_*‐weighted MRI performed with Lac‐FcMOF on H22‐tumor bearing mice, with images taken before and 12 h after intratumoral injection (Figure S17c, Supporting Information), showed strong negative contrast, which indicates that Lac‐FcMOF disperses well within the tumor following intratumoral injection and can be an effective MRI contrast agent for guiding MMHT.

### 2.8 In Vivo Magnetothermal Efficiency

Different concentrations of Lac‐FcMOF were injected into the tumors of H22 tumor‐bearing mice to investigate its magnetothermal effect in vivo. As shown in Figure 5d and e, the temperature increase in the PBS group after 20 min of AMF was 2.0 °C, which was likely caused by thermal radiation of the induction coil. With the increase in Lac‐FcMOF concentration, more rapid and elevated temperature increase at the tumor site was observed. Intratumoral injection of 50 µL 20.0 mg mL^−1^ Lac‐FcMOF and treatment with AMF for 20 min increased the temperature of the tumor site by 8.2 °C, which indicates that Lac‐FcMOF is suitable for MMHT.

### 2.9 In Vivo Anti‐Tumor Efficacy

**Figure 6:**
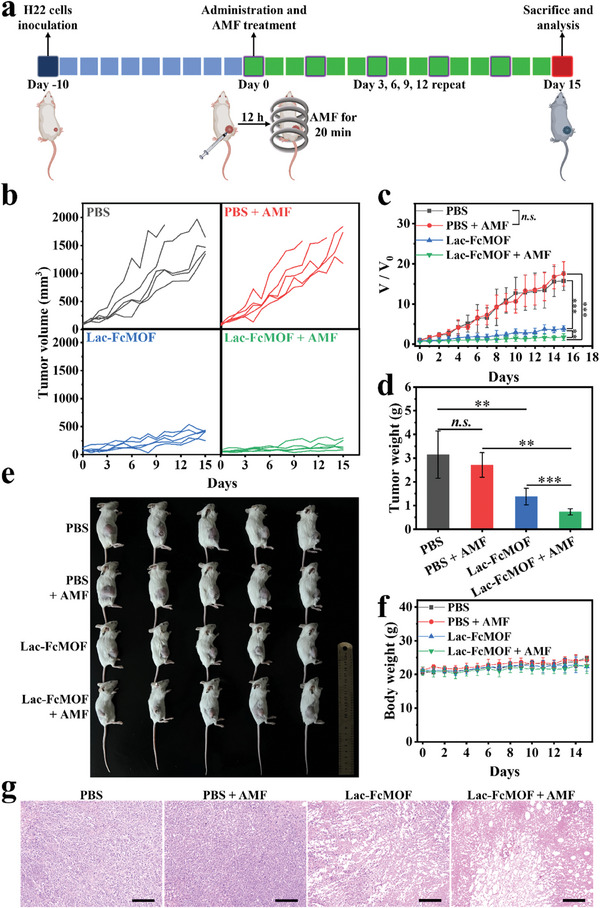
In vivo anti‐tumor efficiency. a) Treatment timeline for H22 tumor‐bearing BALB/c mice. b) Tumor volume changes of each mouse in various treatment groups over time. c) Changes in relative tumor volume in various treatment groups over time. d) Isolated tumor weights across different groups. e) Photograph of sacrificed mice from different groups at 15 d. f) Weight change curve of mice across different treatment groups. Data are shown as the mean ± SD (n = 5) (* *p* < 0.05, ** *p* < 0.01, *** *p* < 0.001 and n.s. denotes not significant). g) H&E‐stained tumor section images from each group of mice. Scale bar: 200 µm. Section thickness: 5 µm.

Encouraged by the enhanced anti‐cancer properties of Lac‐FcMOF observed in vitro, in vivo efficacy experiments were conducted using H22 tumor‐bearing mice (**Figure** **6**a). H22 cells are widely used murine hepatic cancer cells that overexpress ASGPRs, and are suitable for constructing immune‐compromised murine hepatocarcinoma tumor‐bearing models.^[^ ^67^, ^68^, ^69^ ^]^ Mice were divided into four groups (n = 5 each) that received different treatments, including PBS, PBS + AMF, Lac‐FcMOF, and Lac‐FcMOF + AMF. As shown in Figure 6b–e, mice in the PBS and PBS + AMF group showed similar trend in tumor volume, tumor weight and growth, indicating that treatment with AMF alone had no effect on tumor growth. In contrast, the group treated with Lac‐FcMOF displayed effective inhibition of tumor growth and tumor weight. Furthermore, the Lac‐FcMOF + AMF group showed even greater tumor growth suppression and tumor weight reduction, leading to a tumor inhibition rate of 90.4%. Extended survival time after treatment with Lac‐FcMOF and Lac‐FcMOF + AMF was also observed, as shown by the survival curve in Figure S18 (Supporting Information). Additionally, mice in all groups exhibited gradual weight gain during treatment and no notable distinction in body weight (Figure 6f). Finally, tumors and organs were collected and stained with hematoxylin and eosin (H&E) to assess the anti‐tumor effectiveness and potential systemic toxicity of various treatments. Clear tumor tissue damage could be observed in the Lac‐FcMOF and Lac‐FcMOF + AMF groups with no observable damage to major organs (Figure 6g; Figure S19, Supporting Information). All these results suggest that Lac‐FcMOF‐mediated enhanced MMHT exhibits excellent tumor inhibition ability as well as good biocompatibility in vivo.

## 3 Conclusion

In summary, a lactose derivative modified paramagnetic FcMOF was developed for the first time to enhance the therapeutic efficacy of MMHT by downregulating HSPs and promoting ferroptosis through the aggravation of two‐way regulated RDH. Lac‐FcMOF has a uniform nanosize with a porous structure and exhibits magnetic hyperthermia property and thermal stability. In vitro experiments demonstrated that Lac‐FcMOF effectively targets HepG2 cells, and aggravates two‐way regulated RDH, which can be further enhanced via its magnetothermal effect. This results in HSP70 downregulation and ferroptosis, which synergizes with MMHT to induce more efficient tumor cell death. In vivo experiments demonstrated that Lac‐FcMOF exhibits excellent biocompatibility and anti‐tumor activity with a tumor inhibition rate of 90.4%. This work offers a new strategy for the simple construction of a magnetic hyperthermia agent that is capable of downregulating HSPs and inducing ferroptosis by aggravating two‐way regulated RDH for efficient MMHT. Furthermore, compared with ferrites, the porous structure of FcMOF holds far superior drug loading potential, thereby providing the possibility to combine magnetic hyperthermia therapy and various other therapeutic approaches for synergistic tumor treatments.

## 4 Experimental Section

### Materials

The reagents used were of analytical grade, unless otherwise specified, and did not need further purification. Ultrapure water was used in this study. 3‐(4,5‐dimethylthiazol‐2‐yl)−2,5‐diphenyltetrazolium bromide (MTT), sodium methoxide (98%), and CuCl_2_·2H_2_O were bought from Aladdin‐Reagent Co., Ltd. 1,1′‐ Ferrocenedicarboxylic acid, PVP K10, D‐lactose, ethanol, methanol, ethyl acetate, dichloromethane, petroleum ether, glutathione, methylene blue trihydrate (95%), GSH (99%), KBr, boron trifluoride etherate (48%), and 5% Pd/C were purchased from Adamas Chemical Reagent Co., Ltd. DMF, dimethyl sulfoxide (DMSO), and H_2_O_2_ (30%) were purchased from Chengdu Chron Chemical Co., Ltd. Chloroform was purchased from Haohua Chemical Reagent Co., Ltd. NaHSO_3_, NaHCO_3_, Na_2_SO_4_, NaCl, and MgSO_4_ were purchased from Xilong Scientific Co., Ltd. Acetic anhydride and I_2_ were purchased from Sinopharm Chemical Reagent Co., Ltd. 1,2‐bis(2‐chloroethoxy)ethane (98%) and FITC were purchased from HEOWNS Biochem Technologies LLC. 5,5‐Dimethyl‐1‐pyrroline N‐oxide (DMPO, 97%) was purchased from Shanghai Macklin Biochemical Technology Co., Ltd. LBA was purchased from Energy Chemical Co., Ltd. DCFH‐DA, fetal bovine serum (FBS), mitochondrial membrane potential kit (JC‐10 Assay), and reduced GSH assay kit were purchased from Solarbio Science & Technology Co., Ltd. Trypsin was purchased from Leagene Biotechnology Co., Ltd. Penicillin‐streptomycin solution and ATP assay kit were purchased from Beyotime Biotechnology Co., Ltd. Roswell Park Memorial Institute medium (RPMI 1640, Gibco) and Dulbecco's Modified Eagle Medium (DMEM, Gibco) were purchased from Thermo Fisher Scientific, Inc. Lipid peroxide fluorescent probe (Liperfluo) was purchased from Dojindo Molecular Technologies, Inc. Malondialdehyde (MDA) content kit was purchased from Suzhou Michy Biomedical Technology Co., Ltd. Human glutathione peroxidase 4 (GPX4) ELISA kit and human heat shock protein 70 (HSP70) ELISA kit were purchased from Jiangsu Meibiao Biotechnology Co., Ltd. Hepatoma carcinoma (HepG2) cell line, human ovarian cancer (SKOV3) cell line, human colon cancer (KM‐12) cell line, human normal liver (HL7702) cell line, human cervical carcinoma (Hela) cell line, human embryonic kidney (293T) cell line and murine hepatoma 22 (H22) cell line were purchased from Jiangsu KeyGEN BioTECH Co., Ltd. Female BALB/c mice (4–6 weeks) were purchased from Hunan SJA Laboratory Animal Co., Ltd.

### Synthesis and Characterization of Lac‐NH_2_

Lac‐NH_2_ was synthesized according to the procedure that was previously reported by the group and the detailed synthetic route was provided in Figures S4 to S8 (Supporting Information).^[^ ^35^, ^70^ ^]^ First, I_2_ (25 mg, 0.1 mmol) and D‐lactose (1.1 g, 3.2 mmol) were added to 40 mL of acetic anhydride and stirred at room temperature for 8 h. After that, the reaction mixture was subjected to extractions with CH_2_Cl_2_ three times. The combined organic phases were then washed three times with saturated NaHSO_3_ solution and saturated Na_2_CO_3_ solution, respectively. The organic phase was subsequently dried over anhydrous Na_2_SO_4_, filtered, and subjected to vacuum distillation to obtain compound **1** (2.0 g, 92% yield). ^1^H NMR (500 MHz, Chloroform‐*d*) *δ* 6.25 (s, 1H), 5.46 (t, *J* = 9.6 Hz, 1H), 5.35 (s, 1H), 5.12 (dd, *J* = 16.5, 7.5 Hz, 1H), 4.98 (dd, *J* = 22.0, 10.5 Hz, 2H), 4.49‐4.43 (m, 2H), 4.17‐4.07 (m, 3H), 4.01‐3.99 (m, 1H), 3.87 (t, *J* = 6.8 Hz, 1H), 3.81 (t, *J* = 9.5 Hz, 1H), 2.17 (s, 3H), 2.15 (s, 3H), 2.12 (s, 3H), 2.05 (s, 9H), 2.00 (s, 3H), 1.96 (s, 3H) ppm. Under N_2_ protection, compound **1** (70 mg, 0.1 mmol) and 1,2‐bis(2‐chloroethoxy)ethane (50 mg, 0.3 mmol) were dissolved in dry CH_2_Cl_2_ (3 mL) and stirred at room temperature for 1 h. Then, at 0 °C, BF_3_·Et_2_O (130 µL, 1 mmol) was added dropwise to the above solution, and the reaction was allowed to proceed for 24 h. After this, the reaction mixture was filtered through celite, and the filtrate was collected and subjected to vacuum distillation. The residue was dissolved in ethyl acetate and washed with saturated NaHCO_3_ and saturated NaCl three times each. The organic phase was dried over anhydrous MgSO_4_, filtered, and then subjected to vacuum distillation. Compound **2** (34 mg, 42%) was obtained after purification by flash column chromatography on silica. ^1^H NMR (500 MHz, Chloroform‐*d*) *δ* 5.34 (dd, *J* = 3.5, 1.2 Hz, 1H), 5.19 (t, *J* = 9.3 Hz, 1H), 5.10 (dd, *J* = 10.4, 7.9 Hz, 1H), 4.95 (dd, *J* = 10.4, 3.5 Hz, 1H), 4.89 (dd, *J* = 9.6, 7.9 Hz, 1H), 4.57 (d, *J* = 7.9 Hz, 1H), 4.51‐4.44 (m, 2H), 4.15‐4.06 (m, 3H), 3.93‐3.85 (m, 2H), 3.81‐3.79 (m, 1H), 3.77‐3.59 (m, 12H), 2.15 (s, 3H), 2.12 (s, 3H), 2.06 (s, 3H), 2.04 (s, 9H), 1.96 (s, 3H) ppm. Thereafter, compound **2** (39 mg, 0.05 mmol) and NaN_3_ (16 mg, 0.25 mmol) were dissolved in DMF (3 mL) and reacted overnight at 80 °C. CH_2_Cl_2_ was then added to dilute the reaction mixture, followed by washing three times with ultrapure water and saturated NaCl solution, respectively. The organic phase was dried over anhydrous MgSO_4_, filtered, and then subjected to vacuum distillation. Compound **3** (30 mg, 77%) was obtained after purification by flash column chromatography on silica. ^1^H NMR (500 MHz, Chloroform‐*d*) *δ* 5.34 (d, *J* = 3.5 Hz, 1H), 5.19 (t, *J* = 9.3 Hz, 1H), 5.10 (dd, *J* = 10.4, 7.9 Hz, 1H), 4.95 (dd, *J* = 10.4, 3.4 Hz, 1H), 4.89 (dd, *J* = 9.5, 7.9 Hz, 1H), 4.57 (d, *J* = 7.9 Hz, 1H), 4.52‐4.42 (m, 2H), 4.15‐4.04 (m, 3H), 3.95–3.84 (m, 2H), 3.79 (t, *J* = 9.4 Hz, 1H), 3.74‐3.69 (m, 1H), 3.68‐3.57 (m, 9H), 3.39 (t, *J* = 5.1 Hz, 2H), 2.15 (s, 3H), 2.11 (s, 3H), 2.06 (s, 3H), 2.04 (s, 9H), 1.96 (s, 3H) ppm. Finally, compound **3** (150 mg, 0.19 mmol) and CH_3_ONa (16 mg, 0.3 mmol) were dissolved in CH_3_OH (2 mL) and allowed to react at room temperature for 2 h. The mixture was neutralized to pH 7.0 by adding a cation exchange resin, filtered, and subjected to vacuum distillation to obtain an intermediate product. The intermediate product was dissolved in CH_3_OH (20 mL), and 5% Pd/C (13 mg, catalyst amount) was then added. The reaction was carried out at room temperature under a hydrogen atmosphere (1 MPa) overnight. The resulting mixture was filtered through celite and subjected to vacuum distillation to obtain Lac‐NH_2_ (77 mg, 86%). ^1^H NMR (400 MHz, D_2_O) *δ* 4.52 (d, *J* = 8.0 Hz, 1H), 4.44 (d, *J* = 7.8 Hz, 1H), 4.11‐4.00 (m, 1H), 3.97 (dd, *J* = 12.3, 2.1 Hz, 1H), 3.91 (d, *J* = 3.4 Hz, 1H), 3.88‐3.56 (m, 17H), 3.53 (dd, *J* = 9.9, 7.7 Hz, 1H), 3.38‐3.32 (m, 2H), 3.21 (t, *J* = 5.2 Hz, 1H) ppm.

All ^1^H NMR spectrum data are align with literature reports.^[^ ^35^ ^]^

### Synthesis and Characterization of FcMOF and Lac‐FcMOF

Nanoscale FcMOF was synthesized according to reported methods with modification.^[^ ^43^, ^44^, ^71^ ^]^ CuCl_2_·2H_2_O (85 mg, 0.5 mmol) and PVP K10 (1.665 g, 15 mmol) were added to 4 mL of DMF and sonicated until completely dissolved, resulting in a pale green clear solution. Then, Fc(COOH)_2_ (55 mg, 0.2 mmol) was added to the above solution and sonicated until completely dissolved, yielding a deep reddish‐brown clear solution. The solution was transferred to a 10 mL Teflon‐lined autoclave and reacted at 150 °C for 24 h. After the reaction, the reaction vessel was left to cool to room temperature, and the reaction solution was then diluted with 4 mL of CHCl_3_ and transferred to a centrifuge tube. The tube was placed in a high‐speed centrifuge to spin at 12 000 rpm for 20 min. After centrifugation, the supernatant was discarded, and the precipitate was washed twice with DMF and CHCl_3_, respectively, and then air‐dried to obtain a black powder, which was stored at −20 °C for future usage. The construction of FcMOF was characterized by FTIR (Vertex70, Bruker, Germany), XRD (D8 ADVANCE A25, Bruker, Germany) and XPS (Thermo Kalpha, Thermo Fisher, USA). The pore distribution, morphology, size, and zeta potential were characterized by automated surface area and porosity analyzer (ASAP2460, Micromeritics, USA), SEM (Nano SEM‐450, FEI, USA), TEM (TECNAI G2 SPIRIT BIO, FEI, USA), and laser nanometer particle size analyzer (ZEN3600, Malvern, UK). The magnetic hysteresis loop was obtained using a vibrating sample magnetometer (LakeShore7404, LakeShore, USA).

To explore the degradation property of FcMOF, 2 mg FcMOF was dispersed in 10 mL of PBS solution with pH 7.4, or pH 5.0, or pH 5.0 + 10 mM GSH, respectively. At 0, 12, 24, and 48 h, hydrodynamic diameter of FcMOF in different conditions was measured using a laser nanometer particle size analyzer. At 48 h, all suspensions were centrifuged at 12 000 rpm for 20 min and the precipitates were washed three times with ultrapure water. The morphology of FcMOF at 48 h under different conditions was characterized using TEM. In addition, at 0, 6, 12, 24, and 48 h, the solution was centrifuged to collect the supernatant, and the relative release of Cu was measured using inductively coupled plasma optical emission spectroscopy (PerkinElmer 8300, PerkinElmer, USA).

To synthesize Lac‐FcMOF, 50 mg FcMOF was added into 10 mL ultrapure water to prepare a 5 mg mL^−1^ FcMOF suspension, labeled as A. 300 mg Lac‐NH_2_ was added into 60 mL ultrapure water to prepare a 5 mg mL^−1^ Lac‐NH_2_ solution, labeled as B. Under room temperature and ultrasonic conditions, A was slowly added dropwise to B and reaction was allowed to slowly continue under ultrasound for 12 h. Then, the mixture was centrifuged at 12 000 rpm for 30 min, the supernatant was discarded, and the precipitate was washed with water three times, and then dispersed in water to obtain the Lac‐FcMOF suspension. Lac‐FcMOF was stored at −20 °C for subsequent use. The construction, morphology, size, zeta potential, and stability of Lac‐FcMOF were characterized by FTIR, TEM, and laser nanometer particle size analyzer.

3 mg FITC was dissolved in 650 µL of DMSO, and then added into 15 mL ultrapure water containing 8 mg Lac‐FcMOF. The mixture underwent 2 h of sonication, followed by 20 h of shaking. After that, the mixture was centrifuged at 16 000 rpm for 30 min, and the resulting precipitate was washed three times with ultrapure water to yield Lac‐FcMOF@FITC for usage in cellular uptake and targeting experiments.

### Magnetothermal Conversion Evaluation

Lac‐FcMOF suspended in 100 µL ultrapure water was prepared at concentrations of 0.2, 0.4, 0.8, and 1.6 mg mL^−1^ in separate 0.5 mL centrifuge tubes. These tubes were then placed within the electromagnetic induction heating coil of an AMF generator (coil diameter: 4 cm, frequency: 548 KHz, output power: 3.8 kW) (HJC‐6, Dongguan Hengjia, China). The temperature variations were monitored using an infrared thermal imager (Ti10, Fluke, USA), and the temperature change curves during a 10 min interval were plotted. Next, a new 0.5 mL centrifuge tube containing 1.6 mg mL^−1^ Lac‐FcMOF suspended in 100 µL ultrapure water was prepared, which was placed within the electromagnetic induction heating coil of the AMF generator (coil diameter: 4 cm, frequency: 548 KHz, output power: 3.8 kW). The temperature changes during five magnetic heating and cooling cycles (heating for 10 min, followed by cooling for 30 min) were monitored with an infrared thermal imager. Additionally, Lac‐FcMOF (20 mg mL^−1^, 100 µL) and BODIPY (100 µg mL^−1^, 100 µL) were injected into the center of pork pieces with different volume (sized 1 × 1 × 1 cm^3^ and 2 × 2 × 2 cm^3^), which were then placed under an alternating magnetic field and exposed to a near‐infrared laser light (685 nm, 1 W cm^−2^), respectively. The surface temperature changes of the pork over 20 min were recorded using a near‐infrared thermal imager. At the end of the experiment, the pork was cut open from the middle to record the internal temperature.

The SLP value was calculated using the following equation^[^ ^5^ ^]^:

(1) $$SLP=\frac{C_{water}V_{sample}}{m_{mag}}\times \frac{\Delta T}{\Delta t}$$

where *C*_*water*_ is the specific heat capacity of water per unit volume, *V*_*sam* *ple*_ is the volume of the Lac‐FcMOF suspension, *m*_*mag*_ is the mass of the magnetic material, and Δ*T*/Δ*t* is the heating rate measured from the first 30 s of heating.

### GSH Consumption and Fenton Catalytic Activity

This experimental procedure was based on methods previously reported with slight modification.^[^ ^61^, ^72^ ^]^ Briefly, 0.5 mM GSH solution was mixed with different concentrations of Lac‐FcMOF. After stirring for various durations (1 h, 4 h, or 10 min) under different conditions, the GSH content was quantified using a reduced GSH content assay kit according to the manufacturer's guidelines. Fenton catalytic activity was characterized through MB degradation experiments and ∙OH capture using a UV–vis spectrophotometer (Shimadzu 1750, Shimadzu, Japan) and an ESR spectrometer (EMXmicro, Bruker, Germany), respectively. For MB degradation experiment, the concentration of MB was 10 µg mL^−1^, Lac‐FcMOF was 100 µg mL^−1^, H_2_O_2_ was 10 mM, and GSH was 10 mM. For ∙OH capture experiment, 10 µL of DMPO was used as a radical trap, which was added into the suspension containing H_2_O_2_ (10 mM) or H_2_O_2_ (10 mM) + GSH (10 mM) + Lac‐FcMOF (100 µg mL^−1^).

### Cell Culture

HepG2, HL7702, and H22 cells used in this work were cultured in Roswell Park Memorial Institute medium (RPMI 1640) with 1% (v/v) antibiotics and 10% (v/v) FBS, and maintained at 37 °C under 5% CO_2_. HeLa, SKOV3, KM‐12 and 293T cells were cultured under identical conditions, except Dulbecco's Modified Eagle Medium (DMEM) was used instead of complete 1640 medium. The culture medium was replaced daily, and sub‐culturing of the cells was performed when they reached 90% confluence.

### Cellular Uptake and Targeting

To evaluate cellular uptake, in each well of a 6‐well plate, 2 × 10^5^ HepG2 cells were seeded and cultured for 24 h. Afterward, 100 µg mL^−1^ Lac‐FcMOF@FITC was added and incubated for 0, 1, 2, 4, 6, and 12 h, respectively. Following the incubation period, the cells were harvested, and the fluorescence intensity at 488 nm was measured using a FCM (CytoFLEX, Beckman Coulter, USA).

To evaluate cellular targeting, in each well of a 6‐well plate, 2 × 10^5^ HepG2, Hela, 293T, and HL7702 cells were seeded and cultured for 24 h. One well of HepG2 cells was pre‐incubated with 2.0 mg mL^−1^ LBA for 4 h, and then 100 µg mL^−1^ Lac‐FcMOF@FITC was added to each well and incubated for an additional 4 h. After the incubation, the cells were collected and the fluorescence intensity at 488 nm was measured using a FCM. Three replicate wells were established for each experimental group.

### Cytotoxicity Evaluation

Cytotoxicity against HepG2, SKOV3, Hela, KM‐12 and HL7702 cells was evaluated using MTT assays. Cells were seeded into 96‐well plates with complete 1640 or DMEM medium (10^4^ cells mL^−1^, 100 µL). After 24 h, the medium was replaced with fresh medium containing different concentrations of FcMOF or Lac‐FcMOF for another 24 or 48 h incubation. The control group was cultured in medium devoid of any sample. Subsequently, the medium was substituted with MTT‐containing medium (0.5 mg mL^−1^). After 4 h, DMSO (100 µL) was added after removing the MTT‐containing medium. The absorbance at 490 nm was measured using a microplate reader (Synergy HT03030923, Bio TeK, USA). Five replicate wells were established for each experimental group.

To evaluate the magnetothermal effect in promoting Lac‐FcMOF cytotoxicity, 2 × 10^5^ HepG2 cells were seeded into 35 mm dishes containing 1.5 mL complete 1640 medium and cultured for 24 h. The culture medium was then replaced with fresh medium or medium containing 400 µg mL^−1^ Lac‐FcMOF, after 12 h, one PBS group and one experimental group were subjected to AMF treatment or 43 °C water bath for 10 min, and then incubated for an additional 12 or 36 h. After that, the culture medium was carefully removed, and the cells were washed three times with PBS. Then, 2 mL of medium containing MTT (0.5 mg mL^−1^) was added to each dish and incubated for 4 h. Subsequently, the MTT‐containing medium was carefully removed, and 2 mL of DMSO was added to each dish, followed by shaking at 25 °C for 10 min. Afterward, 100 µL of the solution was transferred to a 96‐well plate. The absorbance at 490 nm was measured using a microplate reader. Five replicate wells were established for each experimental group.

### Intracellular RDH

To evaluate intracellular GSH, 2 × 10^5^ HepG2 cells were seeded into 35 mm dishes containing 1.5 mL complete 1640 medium and cultured for 24 h. The culture medium was then replaced, and the experimental groups were treated with 400 µg mL^−1^ Lac‐FcMOF for 6 h. Before the end of the incubation, one experimental group underwent a 10 min AMF treatment. The intracellular GSH was measured following the instructions provided by the manufacturer of the reduced GSH assay kit. Three replicate wells were established for each experimental group.

To evaluate intracellular ROS, DCFH‐DA was used as a ROS probe. HepG2 cells were seeded into 35 mm plastic bottomed µ‐dishes containing 1.5 mL complete 1640 medium and at a density of 10^5^ cells dish^−1^ cultured for 24 h. The culture medium was then replaced, and the experimental groups were incubated with 400 µg mL^−1^ Lac‐FcMOF for 6 h. Before the end of the incubation, one experimental group underwent a 10 min AMF treatment. After the incubation, DCFH‐DA was added for 20 min incubation. The probe was removed, and images were captured using CLSM (STELLARIS 8 F03040105, Lecia, Germany). The mean fluorescence intensity was analyzed by ImageJ.

### Mitochondria Damage and Intracellular HSP70 Evaluation

To evaluate mitochondrial membrane potential change, JC‐10 was used as a detection probe. HepG2 cells were seeded into 35 mm plastic bottomed µ‐dishes containing 1.5 mL complete 1640 medium at the density of 10^5^ cells dish^−1^ and cultured for 24 h. The culture medium was then replaced, and the experimental groups were incubated with 400 µg mL^−1^ Lac‐FcMOF for 6 h. Before the end of the incubation, one experimental group was subjected to AMF treatment for 10 min. After the incubation, the culture medium was carefully removed, and the procedure outlined in the manufacturer's instructions for mitochondrial membrane potential kit (JC‐10 Assay) was followed. Subsequently, images were acquired using CLSM. To detect intracellular ATP content, the cell culture and treatment procedures were the same as that for intracellular GSH detection, but using the ATP Assay Kit instead.

To evaluate intracellular HSP70, HepG2 cells were seeded into 35 mm dishes containing 1.5 mL complete 1640 medium at a density of 2 × 10^5^ cells dish^−1^ and cultured for 24 h. The culture medium was then replaced, and the experimental groups were incubated with 400 µg mL^−1^ Lac‐FcMOF for 6 h. Before the end of the incubation, one PBS group and one experimental group were subjected to AMF treatment for 10 min, and one PBS group and one experimental group were subjected to 43 °C water bath for 10 min. The HSP70 content was measured following the guidelines provided by human heat shock protein 70 (HSP70) ELISA kit.

### Ferroptosis Related Markers

To evaluate intracellular LPO, Liperfluo was used as an LPO probe. The cell culture and treatment procedures were the same as the evaluation of intracellular ROS. For evaluating intracellular MDA and GPX4, the procedure of cell culture and treatment were the same as for intracellular GSH detection, but using the malondialdehyde (MDA) content kit and human glutathione peroxidase 4 (GPX4) ELISA kit instead.

### Animal Models

All animal care and handling protocols have been approved by the Animal Care and Use Committee of Northwest A&F University (Shaanxi, China) (NWAFU‐314020038). Research methods involving animals were conducted according to approved guidelines. H22 cells (5 × 10^6^) suspended in 100 µL of PBS were injected subcutaneously into the right hind leg of mice. Treatments began when the tumors volume reaches 50–100 mm^3^. The volume of tumors was calculated according to the formula: Volume (mm^3^) = (width^2^ × length)/2.

### In Vitro and in Vivo MRI

Longitudinal relaxation time (*T_1_*) value and transverse relaxation time (*T_2_*) value of an aqueous solution with different concentrations of Lac‐FcMOF were measured on a 0.52 ± 0.05 T clinical MRI scanner (MiNiMR60‐Analyst, China) at room temperature. The *T_2_*‐weighted MRI of H22‐tumor bearing mice before and 12 h after intratumoral injection of Lac‐FcMOF (50 µL, 20 mg mL^−1^) was acquired using a fast spin three echo sequence with the following parameters: T_2_R/T_2_E = 5500/20 ms, NE = 16, FOV = 256 × 256 matrices, repetition time = 2, slice thickness = 2 mm).

### In Vivo Magnetothermal Effect

A 50 µL PBS suspension of Lac‐FcMOF at different concentrations (0, 10.0, and 20.0 mg mL^−1^) was injected into the central part of the tumor. After 12 h, the mice were euthanized and placed into the electromagnetic induction heating coil of an AMF generator at room temperature for 20 min, during which the tumor temperature was measured using an infrared thermal imager every 2 min and infrared thermal images were taken every 10 min. The captured images were analyzed using the SmartView Classic software.

### In Vivo Anti‐Tumor Efficacy

Mice were randomly divided into 4 groups with 5 mice per group: PBS group, PBS + AMF group, Lac‐FcMOF group, and Lac‐FcMOF + AMF group. The administration was conducted via intratumoral injection, where the PBS group and PBS + AMF group received a respective injection of 50 µL PBS while the Lac‐FcMOF group and Lac‐FcMOF + AMF group received a respective injection of 50 µL Lac‐FcMOF (20 mg mL^−1^). After 12 h, the mice in the AMF groups were placed into electromagnetic induction heating coil of an AMF generator and subjected to continuous heating at room temperature for 20 min. Each group received treatment every 3 days, with a total of 5 consecutive treatments. Tumor volume and mouse weight were assessed daily during the study, and the survival curves were also recorded. On the 15th day after initiating the therapy, the mice were euthanized. Organs and tumors were harvested, and tumor weights were documented. Pictures of the tumors were also collected.

### Histology Analysis

The collected tumor tissues and major organs were fixed in Bouin's fixative buffer for 24 h to ensure optimal preservation. Subsequently, paraffin section examination was conducted for histological analysis. Xylene was employed for efficient deparaffinization of the sections, followed by subsequent rehydration using 100%, 95%, and 70% alcohol solutions. Finally, Hematoxylin and Eosin (H&E) staining was applied to the sections to reveal the cellular morphology and structure. The resulting images were captured using a fluorescence microscope (DM5000 B, Leica, Germany) to facilitate analysis.

### Statistical Analysis

All data were presented as the mean ± standard deviations (SD) from at least three repeated experiments. The samples were statistically analyzed using a two‐tailed Student's t‐test and one‐way analysis of variance (ANOVA). *p* < 0.05 is considered significantly different, the significance levels are *p < 0.05, **p < 0.01, ***p < 0.001, and n.s. denotes not significant. The Graphpad Prism 8.0.1 was used for data statistics.

## Conflict of Interest

The authors declare no conflict of interest.

## Supporting information

Supporting Information

## Data Availability

The data that support the findings of this study are available in the supplementary material of this article.
