# Supplementary material for: A Paramagnetic Metal‐Organic Framework Enhances Mild Magnetic Hyperthermia Therapy by Downregulating Heat Shock Proteins and Promoting Ferroptosis via Aggravation of Two‐Way Regulated Redox Dyshomeostasis
Source: Adv Sci (Weinh). 2023 Dec 31;11(11):2306178. doi: 10.1002/advs.202306178 (PMC10953551; doi:10.1002/advs.202306178)
Supplement: Supplementary file 1 — Supporting Information [file ADVS-11-2306178-s001.pdf]

ADVANCED SCIENCE
Open Access

Supporting Information

for *Adv. Sci.*, DOI 10.1002/advs.202306178

A Paramagnetic Metal-Organic Framework Enhances Mild Magnetic Hyperthermia Therapy by Downregulating Heat Shock Proteins and Promoting Ferroptosis via Aggravation of Two-Way Regulated Redox Dyshomeostasis

Yi Wang, Zelong Chen, Jiahui Li, Yafei Wen, Jiaxuan Li, Yinghua Lv, Zhichao Pei and Yuxin Pei**

A Paramagnetic Metal-Organic Framework Enhances Mild Magnetic Hyperthermia Therapy by Downregulating Heat Shock Proteins and Promoting Ferroptosis via Aggravation of Two-Way Regulated Redox Dyshomeostasis

Yi Wang, Zelong Chen, Jiahui Li, Yafei Wen, Jiaxuan Li, Yinghua Lv, Zhichao Pei[*]*, Yuxin Pei*[*]

Zhichao Pei, Yuxin Pei

College of Chemistry & Pharmacy

Northwest A&F University

Yangling, Shaanxi 712100, P. R. China

E-mail: peizc@nwafu.edu.cn; peiyx@nwafu.edu.cn

Supplementary Figures

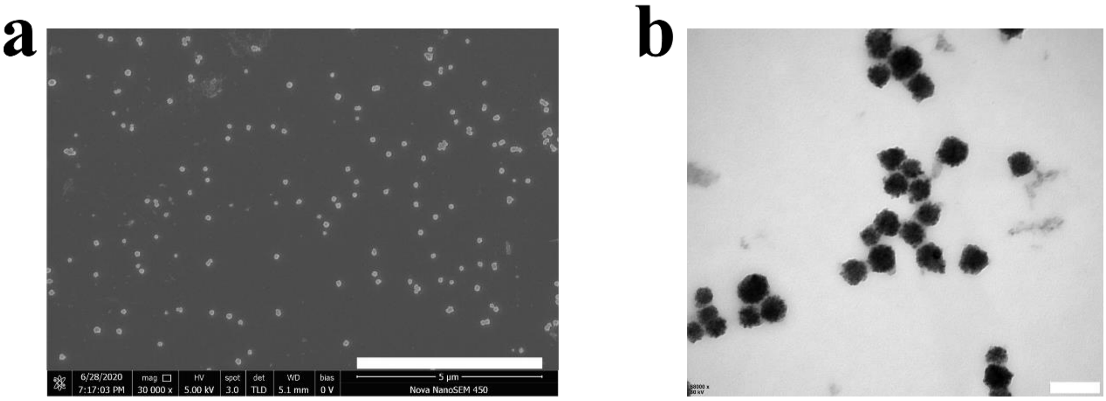

Figure S1. (a) Scanning electron microscope image of FcMOF under a larger field of view. Scale bar: 5 µm. (b) Transmission electron microscopy image of FcMOF under a larger field of view. Scale bar: 200 nm.

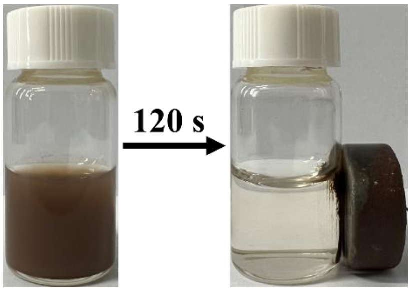

Figure S2. Photos of FcMOF being attracted by a neodymium magnet in water.

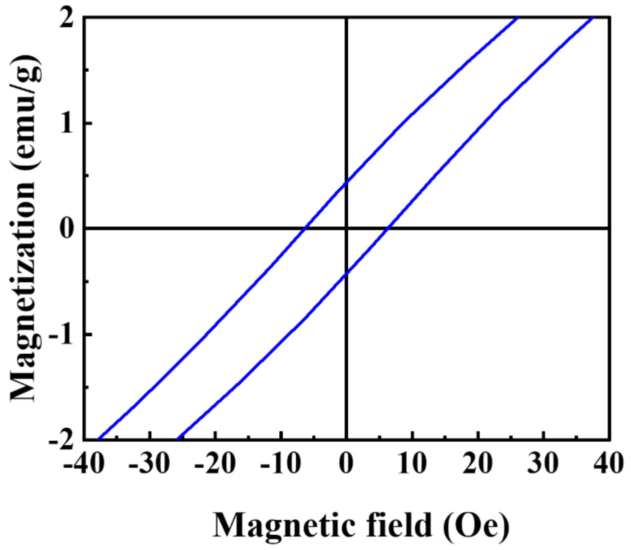

Figure S3. Magnetization hysteresis loop of FcMOF in the range of -40 to 40 Oe.

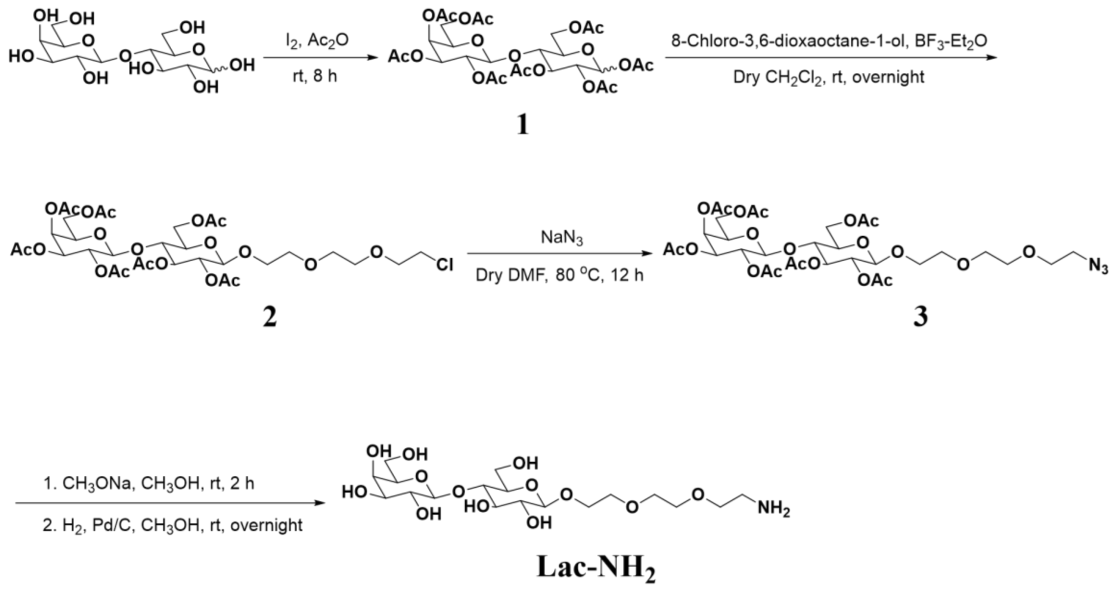

Figure S4. Synthetic route of Lac-NH_2.

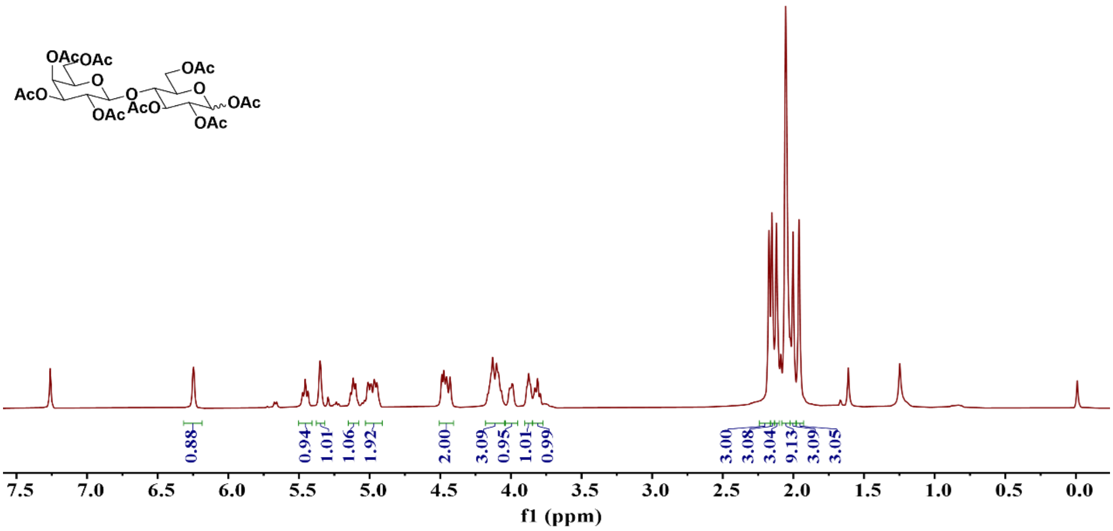

Figure S5. ^{1}H NMR (500 MHz, Chloroform-*d*) spectrum of compound **1**.

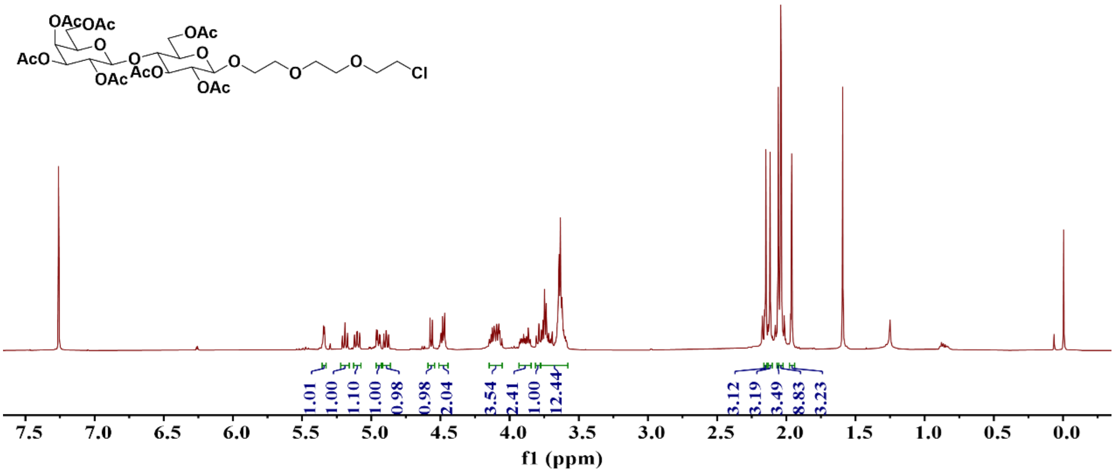

Figure S6. ^{1}H NMR (500 MHz, Chloroform-*d*) spectrum of compound **2**.

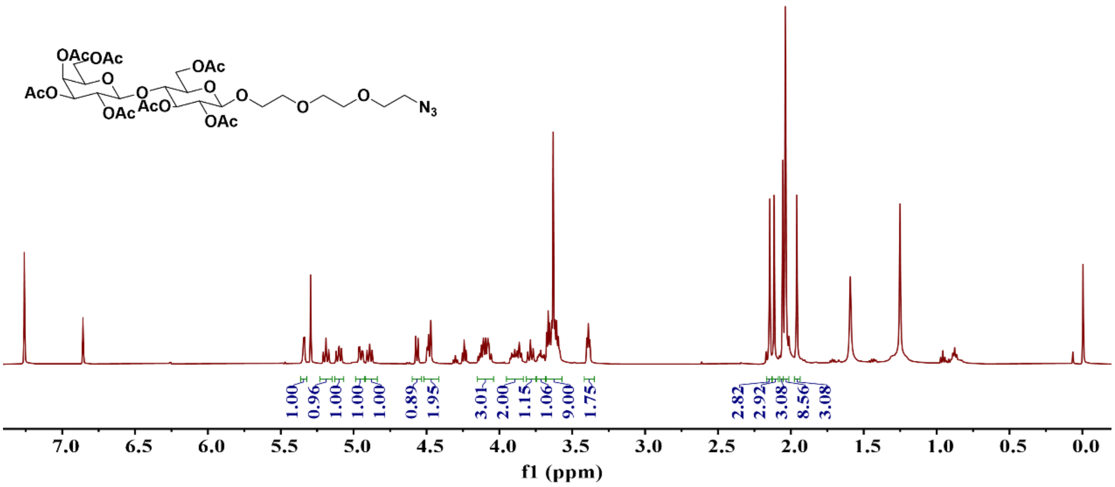

Figure S7. ^{1}H NMR (500 MHz, Chloroform-*d*) spectrum of compound **3**.

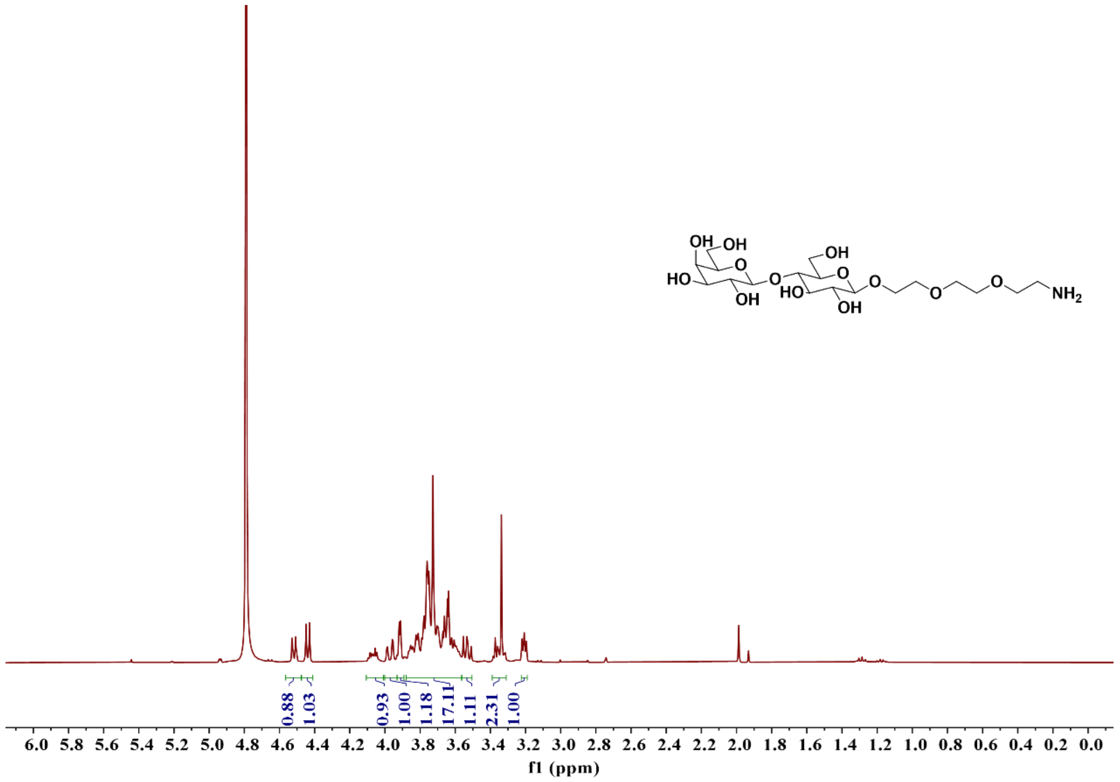

Figure S8. ^1H NMR (400 MHz, D_2O) spectrum of compound Lac-NH_2.

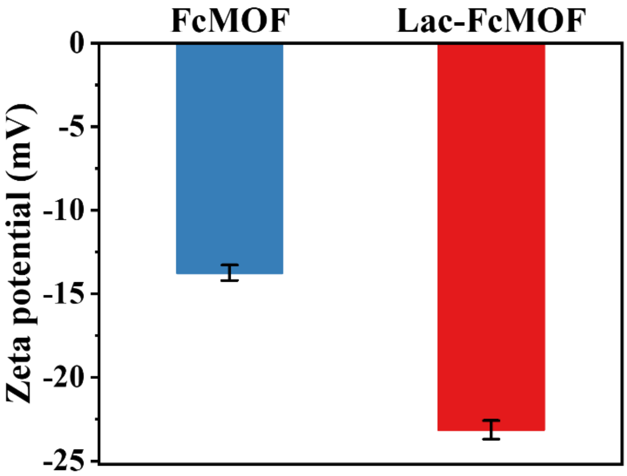

Figure S9. Zeta potential of FcMOF and Lac-FcMOF. Data are presented as the mean ± SD (n = 3).

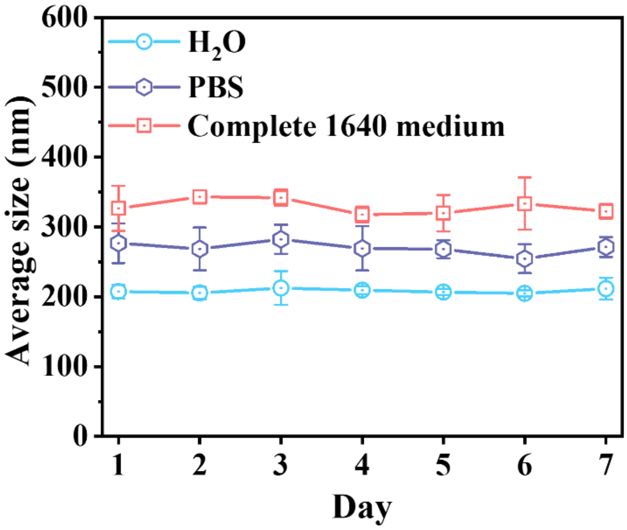

Figure S10. The average hydrodynamic diameter of Lac-FcMOF in water, PBS (pH 7.4) and complete 1640 medium. Data are presented as the mean ± SD (n = 3).

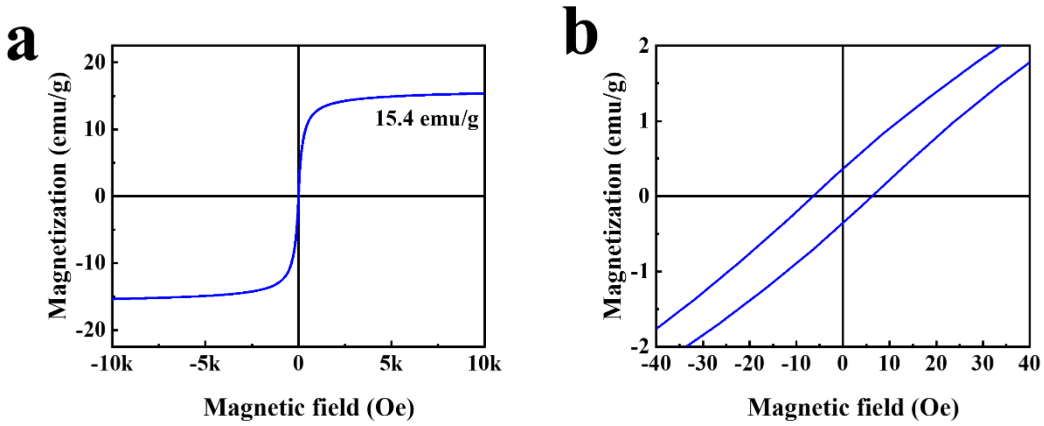

Figure S11. (a) Field-dependent magnetization hysteresis loop of Lac-FcMOF at 300 K. (b) Magnetization hysteresis loop of Lac-FcMOF in the range of -40 to 40 Oe.

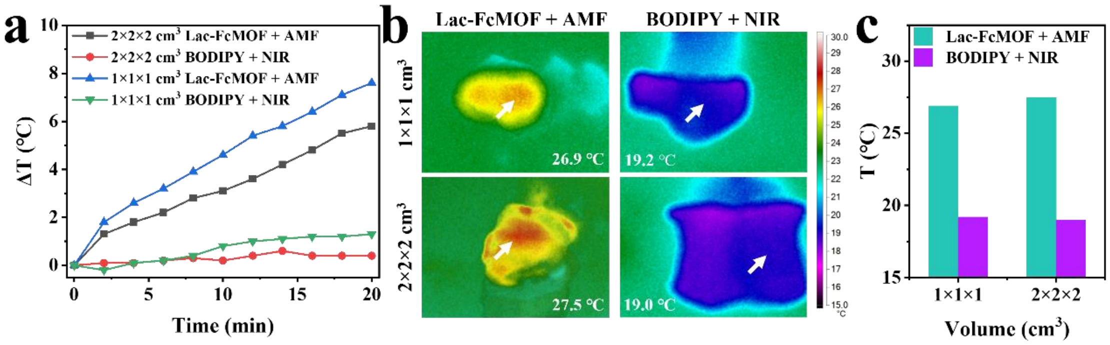

Figure S12. (a) Time-temperature plots of surface of pork pieces after injection with Lac-FcMOF (20 mg/mL, 100 μL) and BODIPY (100 μg/mL, 100 μL), followed by treatment with alternative magnetic field (coil diameter: 4 cm, frequency: 548 KHz, output power: 3.8 kW) and near-infrared (685 nm, 1 W/cm^2) laser light. (b) Infrared thermal images of pork pieces cut from the middle after different treatments. (c) The temperature of each location is indicated by the arrows in (b).

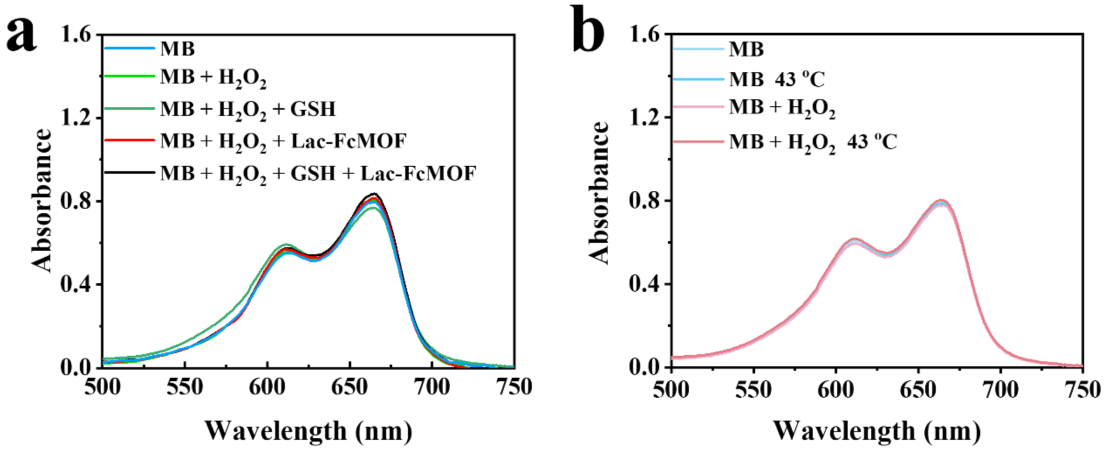

Figure S13. UV-Vis spectra of MB in PBS at pH 7.4 (a) and pH 5.0 (b) after different treatments.

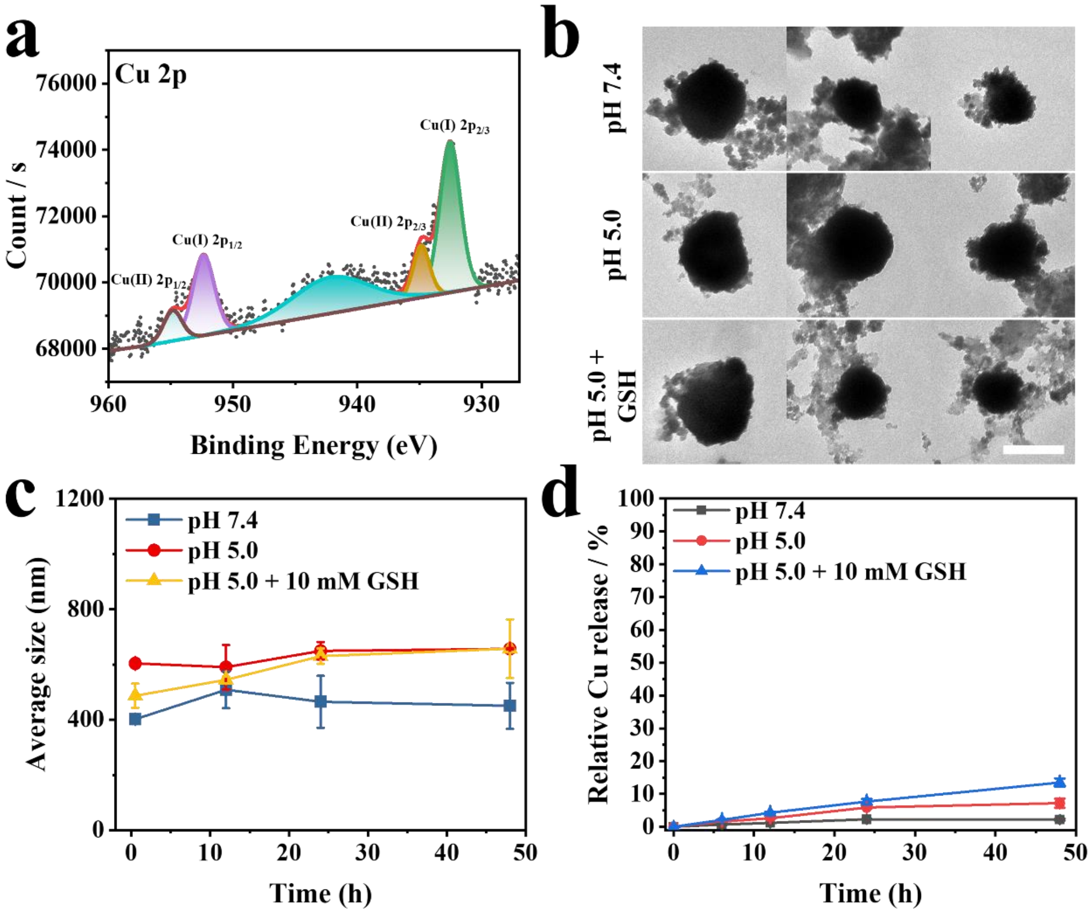

Figure S14. (a) XPS spectra of Cu 2p orbit for FcMOF. (b) TEM images of FcMOF incubated under different conditions for 48 h. Scale bar: 200 nm. (c) The average hydrodynamic diameter of FcMOF under different conditions at different time points. Data are presented as the mean ± SD (n = 3). (d) Relative Cu release from FcMOF under different conditions within 48 h. Data are shown as the mean ± SD (n = 3).

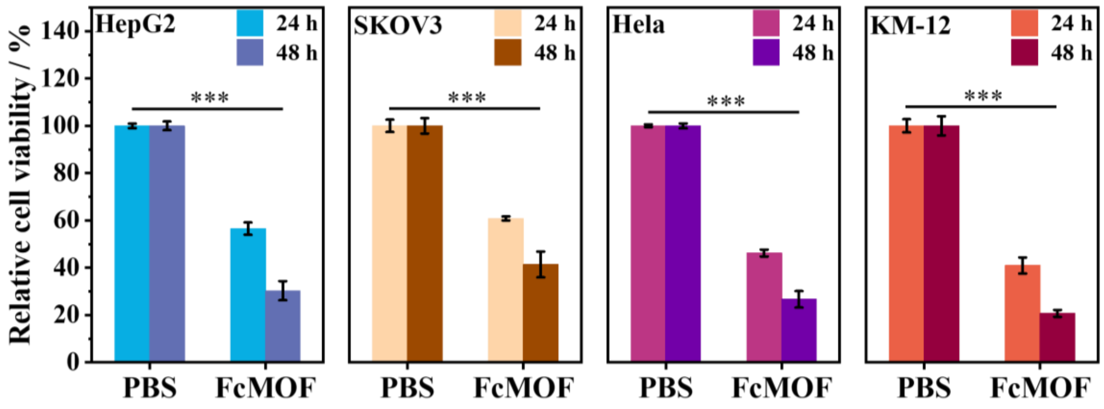

Figure S15. Cytotoxicity of FcMOF (400 μg/mL) towards HepG2, SKOV3, Hela, and KM-12 cells after incubation for 24 h and 48 h. Data are shown as the mean ± SD (n = 5) (*** $p < 0.001$).

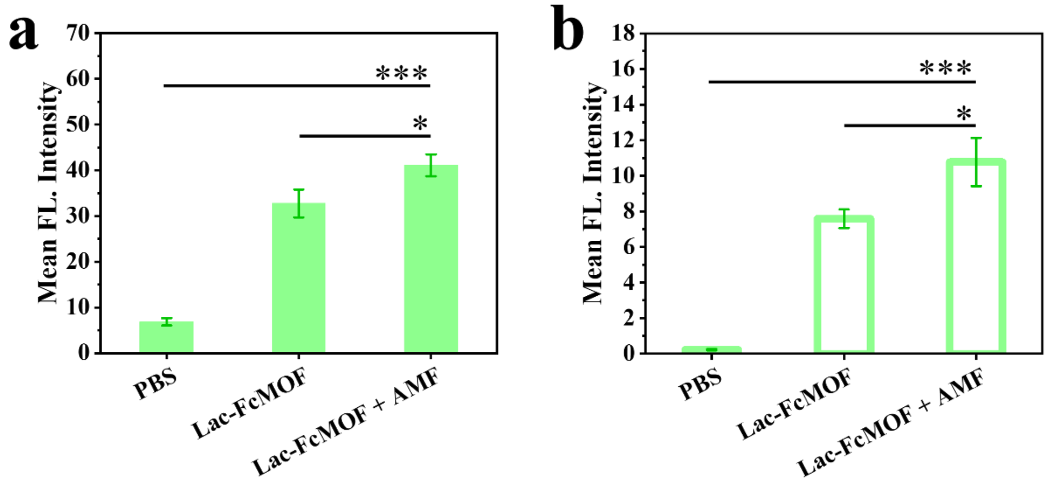

Figure S16. (a) Mean fluorescence intensity of each group in Figure 4b. (b) Mean fluorescence intensity of each group in Figure 5b. Data are presented as the mean ± SD (n = 3) (* $p < 0.05$, and *** $p < 0.001$).

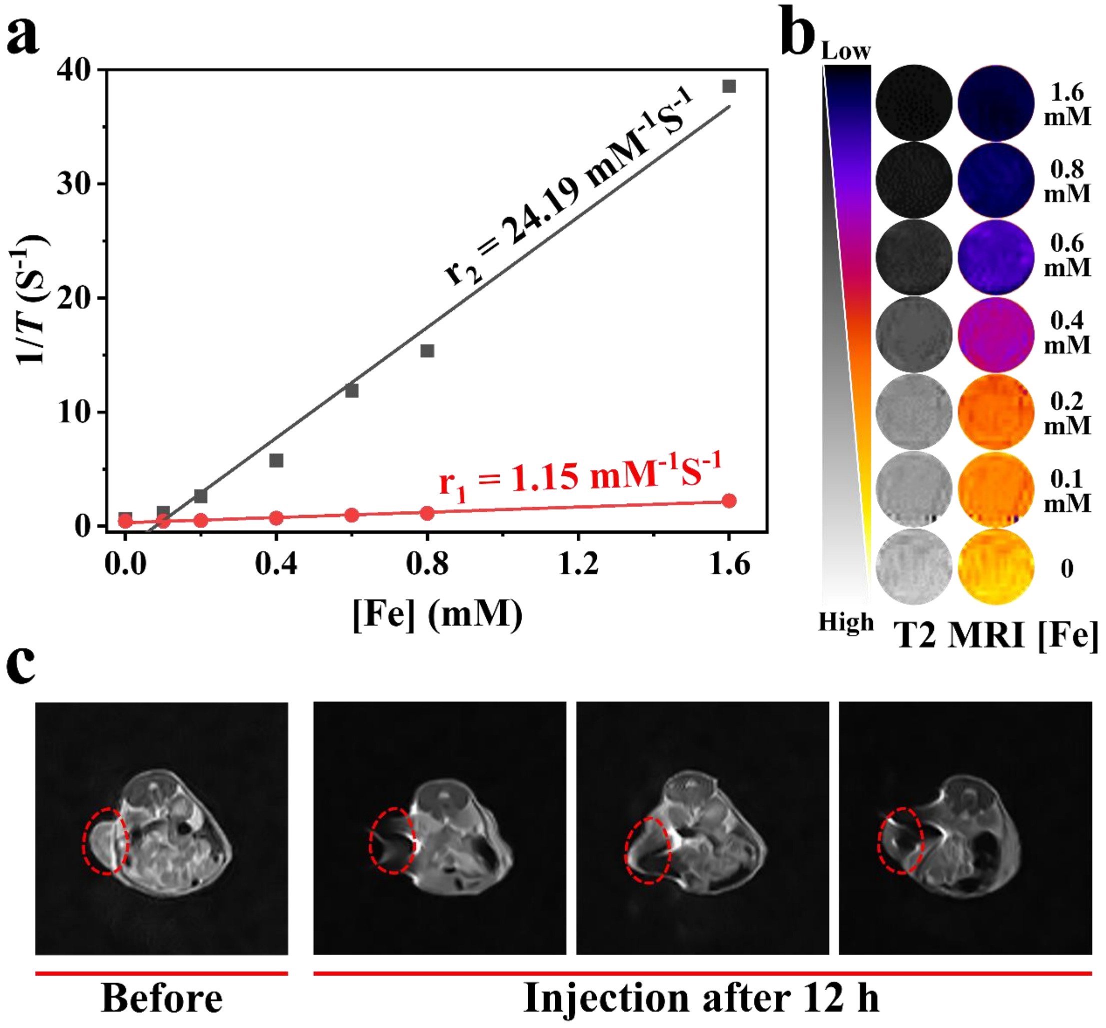

Figure S17. (a) The value of T_1 relaxation rates (r_1) and T_2 relaxation rates (r_2), and (b) T_2-weighted magnetic resonance imaging (MRI) of different concentrations of Lac-FcMOF. (c) T_2-weighted MRI of H22-tumor bearing mice before and injection of Lac-FcMOF after 12 h.

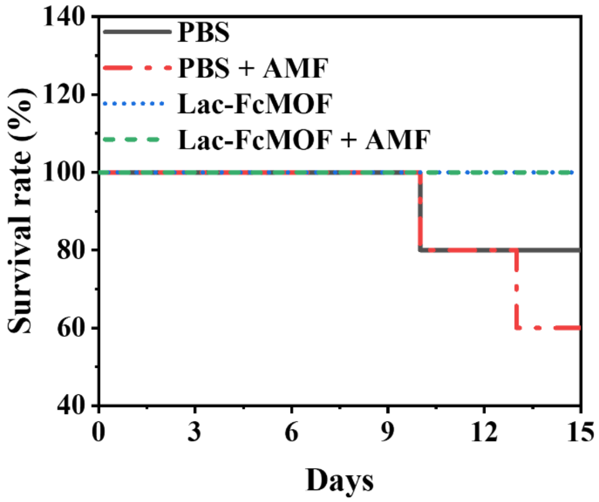

Figure S18. Survival curves of different groups over time.

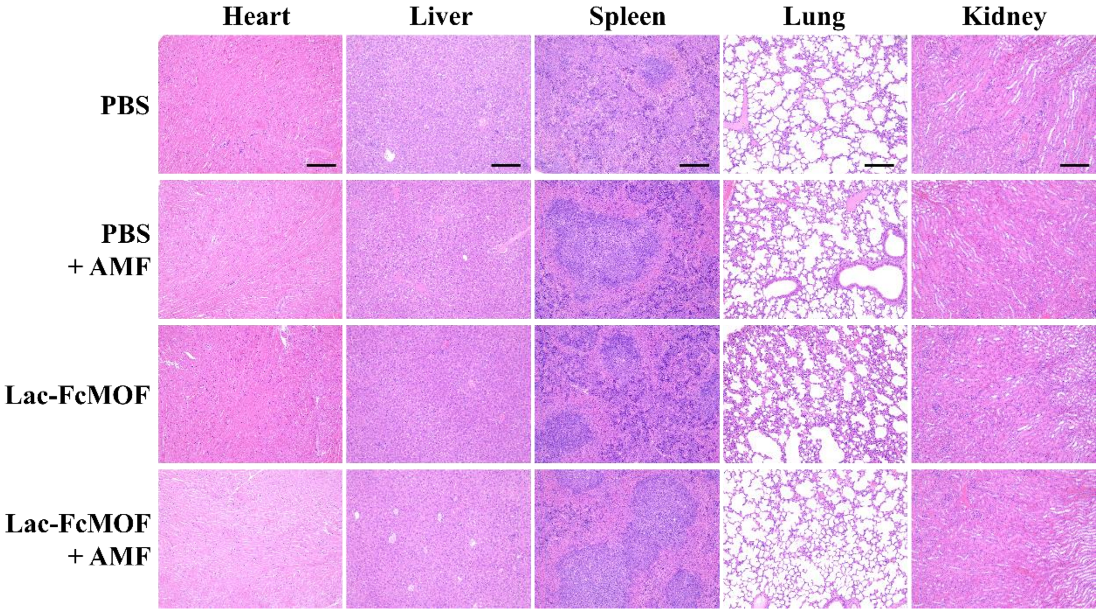

Figure S19. H&E staining of the heart, liver, spleen, lung, and kidney sections of H22 tumor-bearing BALB/c mice following various treatments. Scale bar: 200 μm. Section thickness: 5 μm.
